# Urinary Abscess

**Published:** 1829-04

**Authors:** 


### URINARY ABSCESS.

*Case of Urinary Abscess in the Perineum, treated at* St. George's Hospital.

Adam Davis, æt. fifty-eight, admitted January 27th. He has had stricture for many years, and several surgeons have endeavoured, without success, to pass an instrument into the bladder. A month ago a swelling began to form in the perineum, near the anus, which has gradually increased, but latterly with more rapidity. It at present distends the whole perineum, and extends on the left side to the front of the abdomen, the scrotum and penis being also considerably swollen. In the perineum, fluctuation is easily perceptible, while the scrotum and abdominal boundary of the abscess are hard and tense. He can make water tolerably well, but some usually dribbles away for some time afterwards. He has much constitutional disturbance, with a quick irritable pulse, and anxiety of countenance.

Mr. Hawkins, who had called at the hospital to see another patient, made a free incision in the left side of the perineum, which gave exit to full three-quarters of a pint of urine and pus, with some shreds of sloughy cellular membrane. Some scarifications were also made, to relieve the tension of skin around the abscess. The fingers could be passed freely into an irregular cavity, which was crossed by portions of cellular membrane, which appeared to be in a sloughy state.—℞. Haust. Salin. Ammon. c. Tr. Opii ♏ v. sextis horis. Linseed poultice and fomentation to the wound.

28th.—Very greatly relieved. Tongue moist and clean; pulse much less frequent. Some urine comes by the wound, but the greater part passes through the urethra. The swelling of the scrotum and sides of the nates and perineum is much diminished.

Mr. Hawkins remarked the great difference between a case of the present kind and one of extravasation of urine; a very successful instance of which had recently occurred, under the care of Mr. Keate, the cause being the same in each, viz. irritation, and generally ulceration of the urethra behind the stricture: in the one there being a sudden rupture of the urethra, with a forcible propulsion of a large quantity of urine into the cellular membrane, which, being in a healthy state, is easily lacerated, and allows the urine to pass upwards so as to distend the scrotum and penis, and abdominal parietes: in the other a small quantity of urine only escaping from the urethra, or a small abscess taking place at the side of the urethra, and not containing urine until ulceration had produced a communication with the urethra, and the extension of the abscess being preceded by deposition of lymph into the surrounding textures, so as to form a boundary to the abscess. In the one the infiltration of urine being followed by the most severe constitutional disturbance, with extensive sloughing of the cellular membrane and skin, and by death in a short space of time, unless numerous and extensive incisions are made very early, to afford exit for the sloughs and for the urine: in the other case, the disease proceeding much more slowly, (in the present instance a whole month,) and only so far different from a common abscess as it contains urine mixed with the pus, and requires therefore a free incision low in the perineum, to allow the water to be discharged freely.

29th.—The interior of the abscess is granulating, and the swelling much lessened. Pulse 100, quiet, and rather weak.—℞. Haust. Cinch. Ammon. Carb. gr. iij.; Tr. Opii ♏ v. M. fiat haust ter die sumend.

31st.—The bark having produced diarrhœa, and in consequence of this an increase of his debility, the quantity of laudanum was increased to ♏x. in each dose, and he was ordered a pint of porter daily. An instrument being passed into the urethra, a hard cartilaginous stricture was found, which commenced about four inches down the canal.

February 13th.—The abscess has now nearly closed, except at the wound itself, which is contracting quickly. Much less water passes through the wound, and his health is much improved. A small catgut bougie is passed every other day, which is admitted to a greater depth than could at first be done.

